# A link between synaptic plasticity and reorganization of brain activity in Parkinson's disease

**DOI:** 10.1073/pnas.2013962118

**Published:** 2021-01-11

**Authors:** Diliana Rebelo, Francisco Oliveira, Antero Abrunhosa, Cristina Januário, João Lemos, Miguel Castelo-Branco

**Affiliations:** ^a^Institute of Nuclear Sciences Applied to Health, University of Coimbra, 3000-548 Coimbra, Portugal;; ^b^Coimbra Institute for Biomedical Imaging and Translational Research (CIBIT), University of Coimbra, 3000-548 Coimbra, Portugal;; ^c^Faculty of Medicine, University of Coimbra, 3000-370 Coimbra, Portugal;; ^d^Champalimaud Centre for the Unknown, Champalimaud Foundation, 1400-038 Lisbon, Portugal;; ^e^Neurology Department, Coimbra University Hospital Centre, 3004-561 Coimbra, Portugal

**Keywords:** functional magnetic resonance imaging, positron emission tomography, functional connectivity, molecular imaging

## Abstract

The link between synaptic plasticity and reorganization of brain activity in health and disease remains a scientific challenge. We examined this question in Parkinson’s disease (PD) where functional up-regulation of postsynaptic D_2_ receptors has been documented while its significance at the neural activity level has never been identified. We investigated cortico-subcortical plasticity in PD using the oculomotor system as a model to study reorganization of dopaminergic networks. This model is ideal because this system reorganizes due to frontal-to-parietal shifts in blood oxygen level–dependent (BOLD) activity. We tested the prediction that functional activation plasticity is associated with postsynaptic dopaminergic modifications by combining positron emission tomography/functional magnetic resonance imaging to investigate striatal postsynaptic reorganization of dopamine D_2_ receptors (using ^11^C-raclopride) and neural activation in PD. We used covariance (connectivity) statistics at molecular and functional levels to probe striato-cortical reorganization in PD in on/off medication states to show that functional and molecular forms of reorganization are related. D_2_ binding across regions defined by prosaccades showed increased molecular connectivity between both caudate/putamen and hyperactive parietal eye fields in PD in contrast with frontal eye fields in controls, in line with the shift model. Concerning antisaccades, parietal-striatal connectivity dominated in again in PD, unlike frontal regions. Concerning molecular–BOLD covariance, a striking sign reversal was observed: PD patients showed negative frontal-putamen functional–molecular associations, consistent with the reorganization shift, in contrast with the positive correlations observed in controls. Follow-up analysis in off-medication PD patients confirmed the negative BOLD–molecular correlation. These results provide a link among BOLD responses, striato-cortical synaptic reorganization, and neural plasticity in PD.

## Significance

The association between molecular and functional brain plasticities in health and disease remains an outstanding research challenge. Functional up-regulation of postsynaptic D2 receptors has been documented in PD while its significance at the neural activity level has never been identified. Here we provide a link between synaptic plasticity at the molecular level and reorganization of brain activity patterns. We combined molecular imaging of dopamine D_2_ receptors and fMRI to identify molecular mechanisms underlying functional reorganization in PD. The identification of a relationship between neural activation changes with compensatory molecular phenotypes at the synaptic level paves the way for future work to understand the limits of brain reorganization of functional networks in neurological disorders.

Parkinson’s disease (PD) is a neurodegenerative disorder, triggered by selective loss of dopaminergic neurons in the pars compacta of the substantia nigra and is characterized by hypokinesia, tremor, rigidity, and postural instability (1).

PD offers an opportunity to study the interplay between compensatory functional and molecular mechanisms. The functional significance of putative up-regulation of postsynaptic D_2_ receptors in the basal ganglia (BG) remains a mystery. Such mechanisms are probably best explored at a network level using covariance statistics-based approaches for positron emission tomography (PET) data (2) and investigation of reorganization within well-known circuitry, such as the oculomotor system and its well established cortico-striatal connections.

The BG plays an important role in the generation of motor actions including saccades (3), which are impaired in PD (4). Indeed, reflexive saccades triggered in the direction of a stimulus (prosaccades) (PSs) are mainly hypometric, while voluntary saccades triggered in the opposite direction of a stimulus (antisaccades) (ASs) are often delayed and prone to directional errors (3, 5–7). These findings may be explained by an excessive inhibition of superior colliculus (SC) neurons by the BG and/or decreased preocular motor drive from the frontal cortex through the BG to the SC (8). We have previously demonstrated decreased frontal eye field (FEF) activation in PD (7), in contrast with compensatory activity in parietal eye fields (PEFs).

The saccade network has been well documented in functional magnetic resonance imaging (fMRI) studies. Execution of PSs elicits activation of the FEF, supplementary eye field (SEF), and PEF, while ASs trigger additional activation of the dorsolateral prefrontal cortex and anterior cingulate gyrus (9, 10). PD patients show hypoactivity in FEF, SEF, caudate nucleus, and concomitant relative hyperactivity in parietal areas, during saccade paradigms (7, 11). This leads to the hypothesis that the imbalance between FEF and PEF activation is related with reorganization of striatal connectivity, which we investigated here at molecular and functional levels using covariance statistics.

In physiological conditions, dopamine is known to modulate the frontoparietal eye field circuitry (12–14). Visual representations in posterior areas can be modified by changes in dopamine tone in the prefrontal cortex where both D_1_ and D_2_ receptors subtypes are found in infragranular layers where layer-V FEF neurons project to the SC (11). D_2_ receptor modulation in FEF is more effective when eye movements are performed to the stimulus (PSs) (12).

In this paper, we investigated the reorganization of dopaminergic networks and their relation with functional activation plasticity within the oculomotor network, taking advantage of this well-known circuitry. For molecular neuroimaging we chose the dopamine D_2_ type receptor radioligand ^11^C-raclopride, which is well suited to measure synaptic D_2_ receptors in PD (15–19). In this condition, ^11^C-raclopride imaging often shows a caudal putaminal “tear-drop” reinforcement pattern which is consistent with a putatively compensatory up-regulation of postsynaptic receptors in this area as a response to the nigrostriatal presynaptic impairment (15, 16, 20). In this paper we aimed at identifying a functional correlate of such molecular changes.

We used unimodal and multimodal covariance statistics to explore the relationship between saccade-related fMRI activity and D_2_ receptor volumes of distribution in PD patients. This enabled to identify a relation between postsynaptic dopaminergic status and hemodynamic activity as a function of the oculomotor task. More precisely, first, we investigated the association between the density of receptors (translated as distribution volume ratio [DVR]) in the BG with the density of receptors in areas involved in saccades (FEF and PEF) and, second, the link between DVR in the BG with the blood oxygen level–dependent (BOLD) signal (translated as activation β-weights) of regions involved in saccade execution (FEF and PEF), while performing PS or AS tasks. Furthermore, we investigated whether the same patterns could be identified off medication to rule out these effects being simply explained by medication.

## 1. Materials and Methods

### 1.1 Dataset.

Fourteen volunteers with mild to moderate PD (Hoehn and Yahr 1–3, unified PD rating scale 5–44, six females, mean age ± SD 64.79 ± 5.15, and range 59–73, and for additional clinical and demographic variables see *SI Appendix*, Table S1) were recruited from the movement disorder clinic at Coimbra University Hospital Centre from April 2015 to December 2018. Seven patients (four males and three females) were asked to interrupt their dopaminergic medication (levodopa, dopaminergic agonists, andcatechol O-methyltransferase inhibitors), at least, 12 h before the fMRI experiment (21, 22). We called this subgroup, off-medication PD. The remaining patients performed the fMRI task without suspending their usual dopaminergic medication. All PD patients performed the PET/computerized tomography (CT) examination after suspending their dopaminergic medication, at least, 12 h before the scan.

Nine healthy age-matched controls (four females, mean age ± SD 62.22 ± 4.49, and range 58–68) with no history of neurological, psychiatric, or visual disorder were recruited from our volunteers’ database.

All participants went through cognitive evaluation (minimental state examination [MMSE] and depression assessment, geriatric depression scale [GDS] with 30 items). Exclusion criteria included cognitive deterioration (MMSE < 15 for an illiterate subject; <22, for 1–11 y of education; <27, for >11 y of education), moderate to severe depression (GDS > 21), other types of Parkinsonism and/or inability to perform the oculomotor task inside the fMRI scanner (7).

The study was approved by the Ethics Committee of the Faculty of Medicine of the University of Coimbra, in accordance with the Declaration of Helsinki. All subjects signed the informed consent.

Demographic and clinical features are summarized in *SI Appendix*, Table S1. There was no significant difference among groups in terms of age, gender, cognitive, and mood status.

### 1.2 Study Design.

#### 1.2.1 fMRI experimental protocol.

**Fig. 1.**
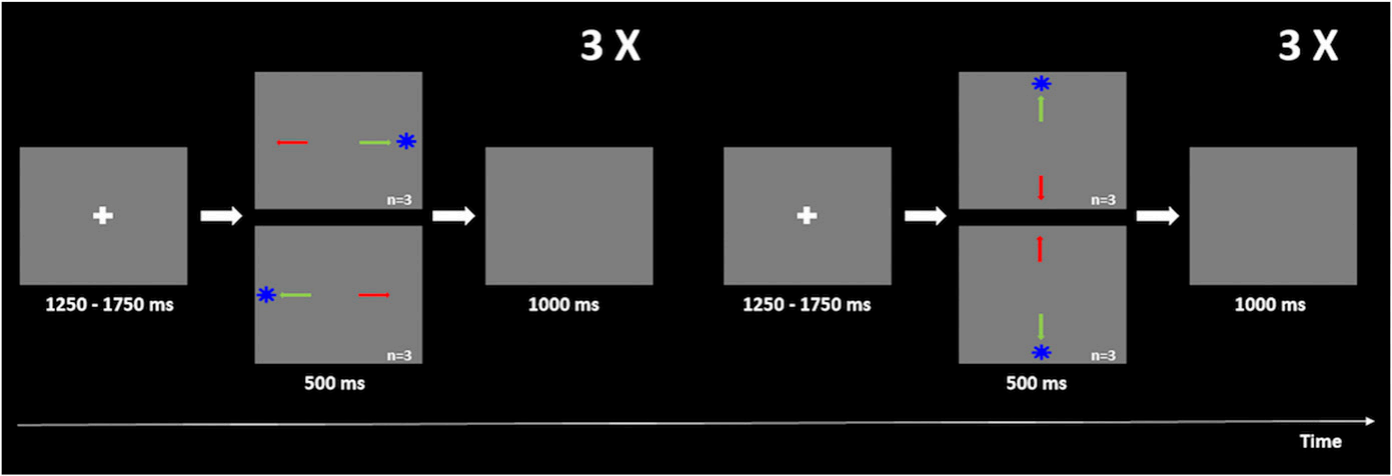
Scheme of the fMRI experimental procedure. For both experiments, participants had to first fixate the white cross displayed in the center of the screen (1,250–1,750 ms total time) and then, toward the blue target (in the PSs task, represented by a green arrow) or in the opposite direction (in the ASs task, represented by a red arrow) quickly and accurately. The target would appear for 500 ms in four possible locations, ordered randomly (10° up, 10° down, 10° right, and 10° left), but organized in blocks of six horizontal saccades interleaved with blocks of six vertical saccades. The trial was completed when the target disappeared and a blank screen was shown for 1,000 ms.

The fMRI experimental procedure (illustrated in Fig. 1) has been described elsewhere (7). Briefly, the stimuli were programed using Presentation software (Version 14.9; Neurobehavioral Systems Inc., CA) and projected with a resolution of 1,024 × 768 pixels, a refresh rate of 60 Hz in a 20 × 15 cm^2^ screen, and 46.5 cm distant from the participant's eyes.

The behavioral task composed two similar paradigms: PSs and ASs. Stimuli were presented against a gray background. Each trial started with a white fixation cross exhibited for 1,250–1,750 ms followed by the appearance of an eccentric blue target appearing in the screen for 0.5 s on one of the four directions (10° down, 10° up, and 10° right or 10° left). Afterward, a blank screen was shown for 1 s, and the trial was completed.

PS and AS runs included 64 trials each. Although random, the presentation of the target was organized in six blocks of horizontal saccades interleaved with six blocks of vertical saccades. Each run began with a 30 s extra period of fixation, and blocks ended with a 16.5 s of fixation to ensure recovery of the hemodynamic response (23).

During the PS run, the participants had to perform a saccade toward the blue target and in the opposite direction in the AS run.

The duration of the fixation cross as well as target positioning was random and counterbalanced.

#### 1.2.2 fMRI acquisition.

Imaging data were collected on a 3.0 T scanner MAGNETOM Trio (Siemens, Erlanger, Germany) using a 12-channel head coil (total acquisition time of 15 min and 25 s per participant). Anatomical volumes were acquired first using a high resolution T1-weighted MPRAGE (magnetization prepared rapid gradient echo) (repetition time = 2,530 ms, echo time = 3 ms, flip angle = 9°; 179 partitions, voxel size = 1 mm^3^, matrix size = 256 × 256, and field of view 256 mm).

Functional scans were obtained using a two-dimensional gradient-echo echo-planar imaging sequence (43 slices, matrix size 86 × 86, field of view 256 × 256 mm^2^, flip angle 90°, voxel size 3 mm^3^, and 91 images). The slices covered the whole brain and were oriented according to a parallel plan regarding the line that passes by anterior and posterior commissures.

#### 1.2.3 fMRI data analysis.

The fMRI data were analyzed using the Statistical Parametric Mapping 12 (SPM version 12) (Wellcome Centre for Human Neuroimaging, Functional Imaging Laboratory, University College London). Standard preprocessing included slice-time correction, realignment, unwarping, anatomical coregistration, segmentation, normalization to the Montreal Neurological Institute (MNI) template, and smoothing with a Gaussian kernel with a full width at half maximum (FWHM) of 6 mm.

The regressors were defined based on the onset and duration of each movement direction (horizontal, which included left and right saccades, and vertical, which included up and down saccades). The regressor called “baseline” represents the time intervals between regressors during which no event occurred. The hemodynamic response was modeled using a canonical response function (24).

To test the main questions of this paper, focused on oculomotor circuitry, a region of interest (ROI)-based approach was performed. The selected ROIs for this study were the left and right FEFs (in Brodmann’s Area [BA] 6/8) (7, 25, 26), the left and right PEFs (in BA 39/40, refs. 7, 27, 28), caudate, and putamen. The last two regions were segmented manually for each participant from PET molecular images and their left and right portions were considered as a single ROI. We opted to segment these regions from distribution volume ratio (DVR) images given the good definition of the ^11^C-raclopride uptake in these structures.

The FEF or PEF ROIs were identified for each subject from functional imaging data: First level contrasts were calculated, and the activated ROIs localized in areas compatible with BA 6/8 or BA 39/40 were segmented (threshold between 2.37 and 5.11 and *P* value between 1 × 10^−6^ and 0.01 with no extended voxels). First level contrasts were computed by setting the regressor of interest to 1 to identify regions responding to horizontal and vertical saccades. Next, per subject, we overlapped the clusters that activated in horizontal and vertical paradigms. Then, we extracted the center of mass of that overlapped region (or peak voxel within the expected BA). The final ROI was defined as a spherical volume with radius of 6 mm. When the intersection was null, we used a mean group ROI-based imputation.

This procedure was performed for PS and AS experiments. Hence, for each participant, we projected six areas of interest (right and left FEFs, right and left PEFs, caudate, and putamen); per ROI and per subject, we extracted the β-weights from fMRI data for each contrast as well as the mean value of the DVR using PET molecular imaging data (see sections 2.2.5 and 2.2.6).

**Fig. 2.**
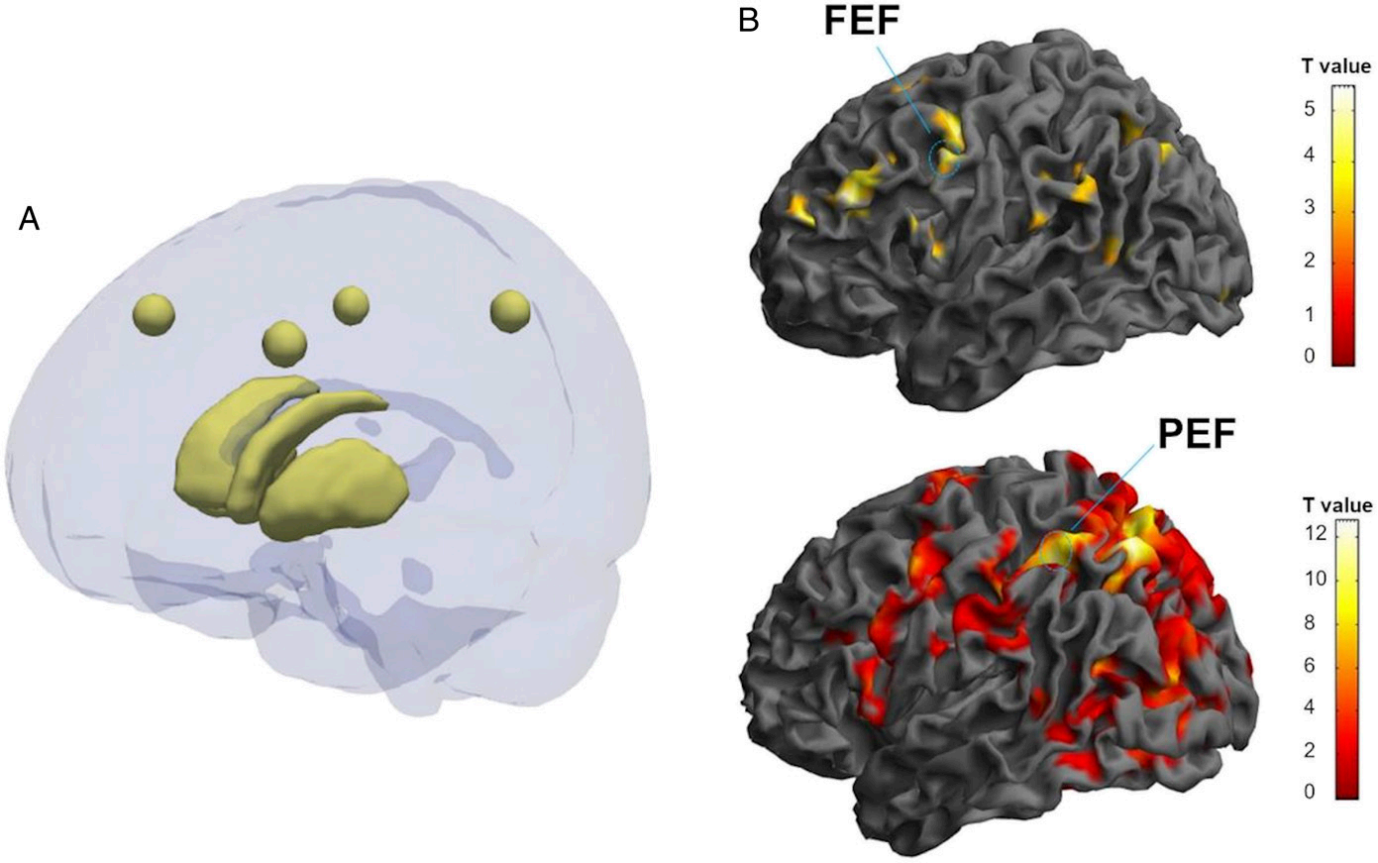
Examples of areas of interest used for further statistical analysis (*A*) Image showing six areas of interest. The spheres correspond to left FEF (center MNI coordinates: −41, 24, and 41), right FEF (center MNI coordinates: 45, 19, and 41), left parietal PEF (center MNI coordinates: −31, −50, and 47), right PEF (center MNI coordinates: 42, −44, and 43). These areas were calculated as a mean ROI from the group in which FEF and PEF clusters, which are consistent with the anatomical literature, as well. Caudate and putamen are represented below. Both areas were segmented manually from molecular imaging data. The image was created using ITK-Snap and Paraview software. (*B*) Examples of functional magnetic imaging statistical activation maps, used to localize the FEF and PEF, and define the ROI masks computed in *A*. A healthy (*Top*) and an on-medication PD participant (*Bottom*) are represented in the figure. In this particular image, we depict the contrast vertical vs. baseline in the PSs experiment for the control subject (MNI coordinates: −54, 4, and 40; *P* = 0.001; T = 3.19) and for the PD patient (MNI coordinates: −32, −50, and 52; *P* = 0.001; T = 3.19). Color bar: t values.

Fig. 2 depicts the six investigated ROIs and statistical fMRI maps, respectively.

#### 1.2.4 PET acquisitions.

All ^11^C-raclopride acquisitions were performed in the same scanner (Philips PET/CT Gemini GXL) preceded by a low-dose brain CT acquisition for attenuation correction. Acquisitions were dynamic, lasted for 90 min (30 frames: 4 × 15 s + 4 × 30 s + 3 × 60 s + 2 × 120 s + 5 × 240 s+ 12 × 300 s), and started immediately after the intravenous bolus injection of ∼555 MBq of ^11^C-raclopride. These were reconstructed using the LOR RAMLA algorithm (Philips PET/CT Gemini GXL) with attenuation and scatter correction. An isotropic voxel size of 2 mm was defined.

*SI Appendix*, Fig. S1 depicts the D_2_ receptor DVR average map in healthy participants.

#### 1.2.5 PET image processing.

The PET dynamic acquisition data were processed to extract the voxelwise DVR using the cerebellum as a reference region. The DVR is the ratio of the radiopharmaceutical DV in a target region with binding sites to that of a reference region (ideally devoid of binding sites). $\text{DVR}=\frac{k_{3}}{k_{4}}+1,$ where $k_{3}$ is the rate constant for transfer from the free to the specific compartment (specific binding of a tracer to a receptor) and $k_{4}$ is the rate constant for transfer from the specific to the free compartment (dissociation rate). Regions with high density of dopamine D_2_ type receptors available have higher DVR than regions with low density of dopamine D_2_ type receptors available. DVR are computed by fitting the following equation to the data extracted from the dynamic PET image (29):

$$\frac{\int_{0}^{t}Target\left(\tau \right)d\tau }{Ref\left(t\right)}=\text{DVR}\left[\frac{\int_{0}^{t}Ref\left(\tau \right)d\tau +Ref\left(t\right)/{\bar{k}}_{2}^{Ref}}{Target\left(t\right)}\right]+int\text{′},$$

where Target(t) and Ref(t) are the concentration over time in the target and reference regions, respectively, and ${\bar{k}}_{2}^{Ref}$ is the mean value of the transference rate from the reference region to the plasma (efflux). The term containing ${\bar{k}}_{2}^{Ref}$ can be neglected since it has no significant influence in the value of the DVR (29). DVR and int′ can be obtained by linear least squares optimization.

In order to overlap FEF and PEF ROIs found using fMRI with PET data to further extract respective DVR values, for each participant, the DVR image (voxelwise DVR) was first rigidly registered with the correspondent MRI-T1 image. Then, the MRI-T1 image was spatially normalized to the MNI space using the DARTEL algorithm implemented in the SPM12, and the geometric transformation found was applied to the DVR image. After this process, the voxelwise DVR of all participants was defined in the MNI space in the same way as the fMRI activation maps. Finally, all normalized DVR images were smoothed with a Gaussian kernel of 12 mm FWHM. Both caudate and putamen were segmented manually using images in the native space.

#### 1.2.6 PET analysis in ROIs derived from fMRI data.

The individual ROIs from FEFs (right and left) and PEFs (right and left) found functionally from fMRI data analysis and, thus, that derived from PS and AS experiments and the manually segmented ROIs of caudate and putamen were used as masks over the spatially normalized DVR images. Using the 3D Slicer Software, the mean DVR for each region and for each subject was extracted for further statistical analysis.

#### 1.2.7 Statistical analysis.

The main goal of this study was to infer about covariance statistics. Such functional connectivity patterns were assessed by calculating the Pearson correlations coefficient between molecular imaging and neural activity and whether they were distinct in PD. To answer our first goal, we investigated unimodal “molecular connectivity” by correlating the mean binding sites from caudate and putamen with the mean binding sites of the right and left FEFs or PEFs. Subsequently, we analyzed multimodal molecular–BOLD activity functional connectivity for the mean binding sites of the caudate or putamen with the BOLD signal represented as β-activation weights from left and right FEFs or PEFs. Direct group comparisons of regression (β) slopes and of differences between *R* values were also performed. The statistical analysis was performed using IBMSPSS vs. 24.

## 2. Results

### 2.1 Uni- and Multimodal Covariance Statistics in the Oculomotor Network and the BG.

The main goals of this paper were to study unimodal (molecular [DVR]–molecular) and multimodal (molecular–functional [BOLD fMRI]) covariance statistics (functional connectivity) patterns in PD across oculomotor regions and the BG. Our main aim was to link the density of receptors in the BG with the saccade evoked BOLD signal in oculomotor regions FEF and PEF. Based on the literature, DVR differences between groups were not expected, which was confirmed in our data set. No previous studies addressed covariance statistics.

#### 2.1.1 Unimodal (molecular–molecular) connectivity.

First, we analyzed the link between DVRs of the caudate and the putamen and DVRs in left or right FEFs/PEFs for the control group and PD groups.

**Fig. 3.**
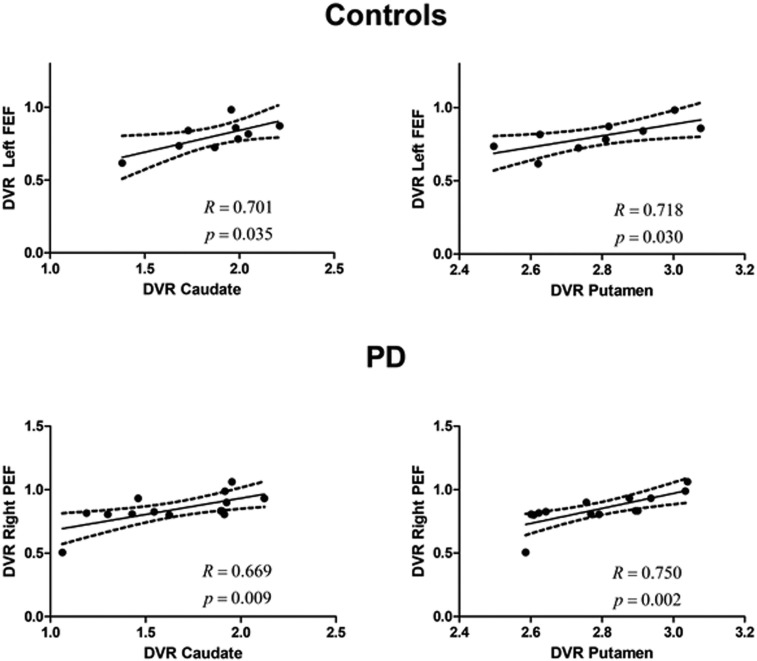
Unimodal (molecular–molecular) correlations in the PSs experiment. The relationship between the DVR in caudate or putamen and the left FEF or right PEF, respectively, in the control group and in the PD group (which included on- and off-medication patients). Regression lines, correlation coefficient, and *P* value for each analysis are presented. The dashed lines represent the 95% confidence band.

In PS-defined ROIs, we found a positive and significant correlation between both the caudate and the putamen and the left FEF in the control group (*R* = 0.701, *P* = 0.035; *R* = 0.718, *P* = 0.030, respectively). Between group slope comparisons showed a significant difference (*t*_19_ = 3.13, *P* = 0.005; *t*_19_ = 2.2, *P* = 0.038, respectively). In the PD group, we found a positive and significant correlation between both the caudate and the putamen and the right PEF (*R* = 0.669, *P* = 0.009; *R* = 0.750, *P* = 0.002, respectively) (Fig. 3). In other words, correlations were FEF dominated in the control group and PEF dominated for the PD group (with both caudate and putamen). This was further confirmed by group comparisons between *R* coefficients: (Left FEF vs. putamen, *Z* = 2.04, *P* = 0.021; left FEF vs. caudate, *Z* = 2.67 *P* = 0.004; right PEF vs. caudate, *Z* = −1.023 *P* = 0.15; right PEF vs. putamen, *Z* = −1.998 *P* = 0.023) (see also *SI Appendix*, Table S2).

**Fig. 4.**
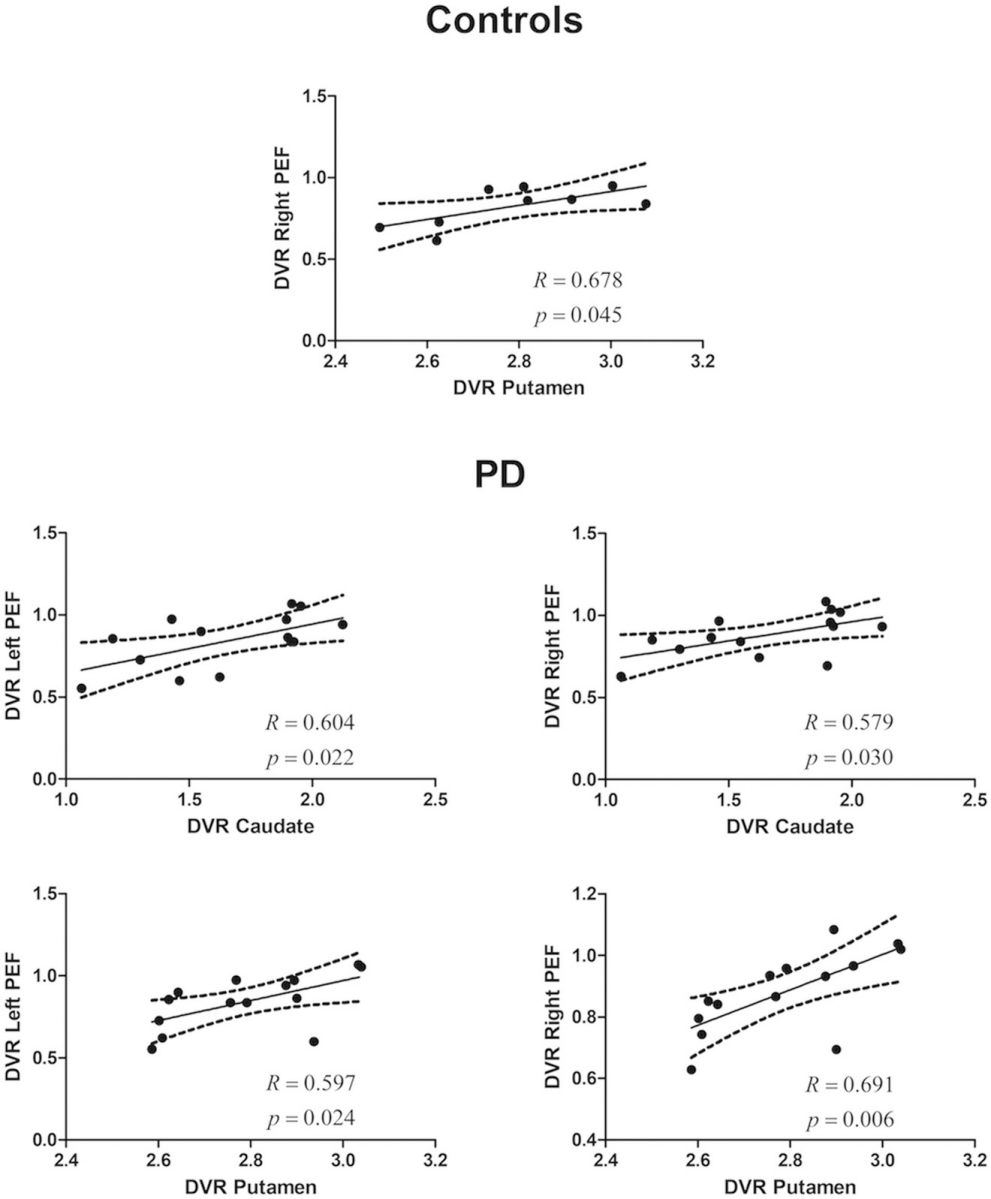
Unimodal (molecular–molecular) in an AS experiment. The relationship (for the AS regions) between the DVR in the caudate or putamen and the left and right PEFs, respectively, in the control group and in the PD group (which included on- and off-medication patients). Regression line, correlation coefficient, and *P* value for each analysis are presented. The dashed lines represent the 95% confidence band.

In AS-defined areas, positive and significant correlations were found between both the caudate and the putamen and the PEF in the PD group (left PEF vs. caudate: *R* = 0.604, *P* = 0.022; right PEF vs. caudate: *R* = 0.579, *P* = 0.030; left PEF vs. putamen: *R* = 0.597, *P* = 0.024; right PEF vs. putamen: *R* = 0.691, *P* = 0.006) (Fig. 4). Concerning controls, we only found significant correlations between the putamen and the right PEF (*R* = 0.678, *P* = 0.045).

#### 2.1.2 Multimodal (molecular–functional) connectivity.

**Fig. 5.**
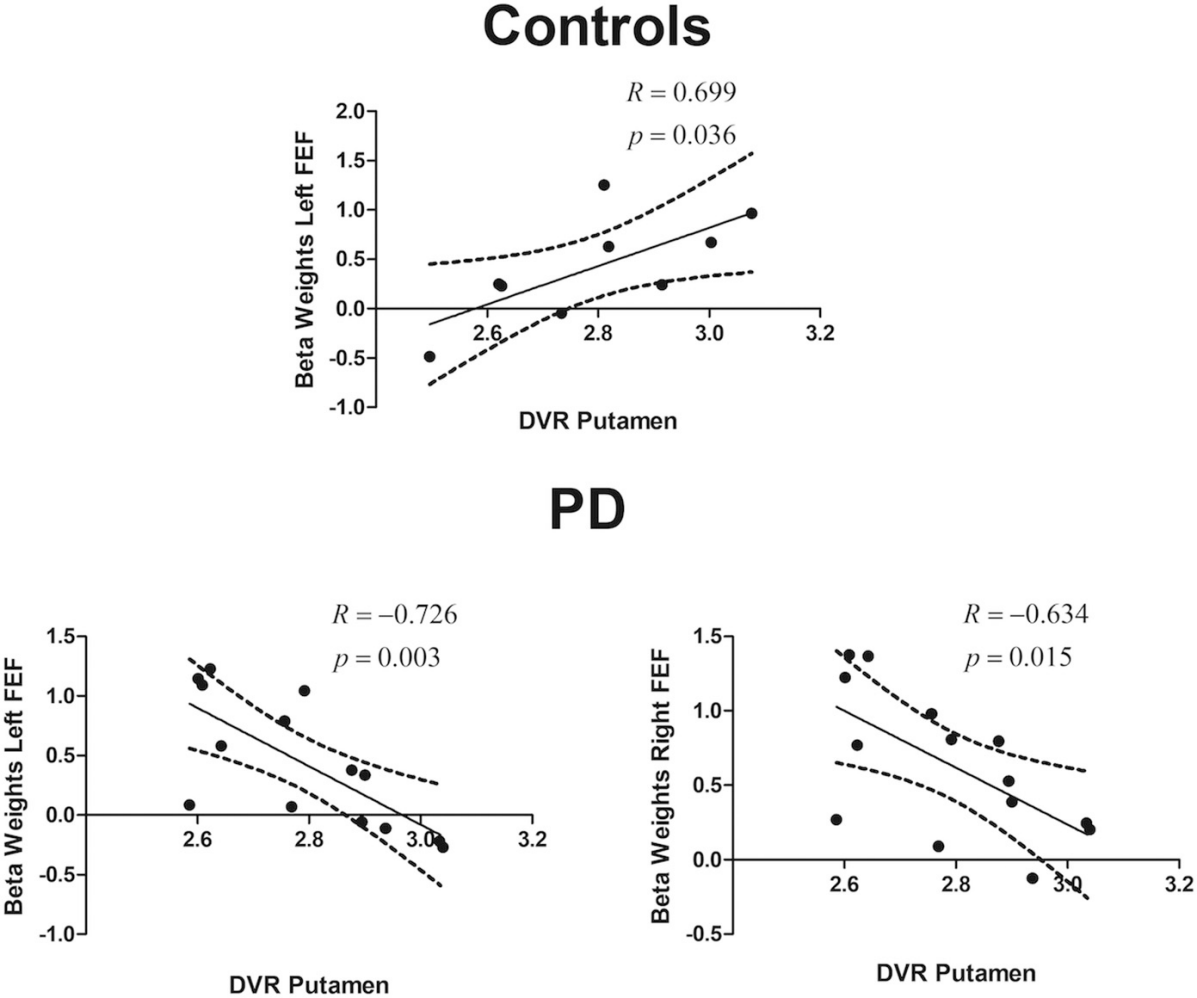
Multimodal molecular–functional correlations in healthy subjects and PD patients. Plot of BOLD fMRI vs. DVR between defined ROIs. The figures depict the relationship between the DVR in the putamen and the β-weights in the left and right FEFs in the control group and in the PD group (which included on- and off-medication patients), while performing vertical PSs. Similar results were found for the right and left FEFs for both groups. Regression lines, correlation coefficient, and *P* value for each analysis are presented. The dashed lines represent the 95% confidence band.

In this section, we focused on molecular (DVR)–BOLD activation correlations (Fig. 5).

Concerning PS-related areas, we found a positive and significant correlation between the DVR in the putamen and the BOLD signal in the left FEF during vertical PS in the control group (*R* = 0.699, *P* = 0.036). In the PD group, we found surprising reversals in polarity of the correlation patterns. Accordingly, we found a negative and significant correlation between the DVR in the putamen and the BOLD signal in the left (*R* = − 0.726, *P* = 0.003) and right FEF (*R* = − 0.634, *P* = 0.015) while performing vertical PS. Fig. 5 shows significant results concerning both the left and the right FEFs. Between group comparisons of difference between (β) slopes further confirmed these effects (putamen—left FEF slope difference, *t*_19_ = 4.37, *P* = 0.0003; putamen—right FEF *t*_19_ = 2.64, *P* = 0.016). Group comparisons between *R* coefficients (after Fisher to *Z* conversion) also showed significant differences (putamen—left FEF, *Z* = 3.518, *P* < 0.0001; putamen— right FEF, *Z* = 2.515, *P* < 0.006).

### 2.2 Disease Effect vs. Medication Effect.

As a follow-up analysis, we investigated a potential medication effect. Concerning PS-related areas, for the off-medication PD group, a negative and significant regression was seen between the DVR in the caudate and the BOLD signal in the left FEF during vertical PS (*R* = − 0.843, *P* = 0.017) and the DVR in the putamen and the BOLD signal in the left FEF during horizontal PS (*R* = − 0.919, *P* = 0.003). *SI Appendix*, Fig. S2 shows the results concerning the PD on- and off-medication groups for the left FEF.

## 3. Discussion

Here we used combined PET/fMRI to test the hypothesis that reorganization of dopaminergic networks is related with functional reorganization of brain activity patterns. We coupled molecular imaging of dopamine receptors with functional neuroimaging using fMRI to study uni- (molecular) and multimodal (molecular–functional) functional correlations (connectivity) patterns. fMRI activation studies in PD have consistently shown activation shifts (11, 17, 30, 31), including a study from our own group suggesting a FEF vs. PEF activity/imbalance in the oculomotor system in PD (7). Such FEF hypoactivation vs. PEF overactivation may be potentially reflected in coupled reorganization at molecular and functional levels. We asked from a functional connectivity point of view whether the molecular phenotype in terms of distribution of D_2_ receptors in striatum relates to redistribution of brain activity in the cortical saccade network. We identified in PD unique unimodal molecular–molecular correlations and most importantly, unique multimodal molecular–functional correlations that showed sign reversal as compared to controls, consistent with the shift model.

When investigating a molecular correlate in PS-related FEF and PEF regions, we found that, while in controls the DVR (binding sites availability) correlated positively between the FEF and the BG, in PD patients, such positive correlation was seen between the BG and the PEF and was absent for FEF. The latter finding suggests favored positive PEF-putamen coupling in PD.

FEF is considered the primary gaze center, providing top-down information to the afferent region PEF to coordinate visual attention (27, 32, 33). Our results may, therefore, impact on predictive coding models where given two distinct and hierarchical regions, impaired processing of the high-level one (34) leads to increased activity in the low-level one as a mechanism to optimize and “accumulate evidence.” Our multimodal pattern of putamen-PEF correlations adds a molecular correlate to such predictive coding models (35).

Thus, the shift from frontal to parietal networks is accompanied by molecular changes as indexed by covariance statistics as markers of synaptic plasticity.

AS-related patterns showed some important distinctions. The more prominent effects observed for PS vs. AS is consistent with the known physiology of D_2_ receptors signaling in the primate frontal cortex whereby D_2_ receptor stimulation affects motor activity tuning only when eye movements are performed to the stimulus (12). The AS task relies distinctly on the BG to hold a reflexive saccade on PEF to calculate the mirror position of the intended saccade and send this information to FEF (36–38). The explicit calculation of such a mirror position may lead to distinct positive correlation patterns. In PD patients, significant correlations with the caudate nucleus were further observed, consistently with the notion that dominant disruption of the putamen in PD may lead to additional recruitment of the caudate.

Importantly, we aimed to rule out if the observed changes—surprising sign reversal in PD patients—in multimodal covariance statistics were due to medication by investigating off-medicated patients. We confirmed again that the positive correlation with FEF was surprisingly reversed in PD patients, who showed a negative correlation also in FEF, in line with circuit reorganization.

The BG exhibit privileged input to FEFs, this way facilitating or inhibiting the execution of saccades (34). Higher FEF activation is associated with better saccade performance in controls, particularly, for more voluntary saccades (8, 10, 39).

However, and as stated above, in PD patients, an inverse correlation was seen: higher binding sites availability in the putamen was negatively associated with levels of BOLD activity associated with the execution of vertical saccades in FEFs. We could replicate this finding in off-medication PD patients. The preserved or greater raclopride putaminal uptake usually seen in PD patients has been ascribed to a generalized loss of endogenous striatal dopamine, leaving dopamine receptors free to bind with raclopride (15, 40). Thus, in patients, contrary to controls, the molecular plasticity reflected in a greater number of putaminal dopamine receptors most probably reflect a compensatory mechanism triggered by permanent dopamine loss in the striatum, which in its turn is associated with a lower hemodynamic response in FEF. Interestingly, our main findings were fully confirmed in off-medication patients. Dopaminergic treatment, on the other hand, seems to ameliorate PSs performance in some but not all studies (41).

To the authors’ best knowledge, no study had performed such uni- and multimodal approaches, providing such a link between molecular and functional reorganization in PD. DVR modifications can be caused by an increase in the number of D_2_ receptors (compensatory denervation hypersensitivity) or by a reduction in extracellular dopamine (the extracellular dopamine reduced by the dopaminergic degeneration can no longer compete for the D_2_ receptor binding with ^11^C-raclopride). We do believe that the second alternative is more unlikely because extracellular dopamine depends on medication, and our effects did show not to depend on medication. Moreover, the link between synaptic dopamine status and reorganization of brain activity patterns as established by PET and fMRI measurements in the same participants would still hold true. Finally, we found striking reversals in regression slopes irrespective of differences in offset levels, which renders the second alternative also unlikely.

Despite the inherent limitations of PET/CT in terms of temporal resolution, the use of covariance metrics has been demonstrated in this regard to be a promising approach (2).

In sum, we found a tight link between functional activation and synaptic changes at the molecular level, reflecting network reorganization in PD. The association between D_2_ receptor binding and reorganization of the saccadic cortical network reflects parietostriatal rerouting in response to a progressive frontostriatal dopamine deficit. This paves the way for future work to understand the limits of brain reorganization of functional networks in neurological disorders.

## Supplementary Material

Supplementary File

## Data Availability

Data available upon request.
