# Supplementary material for: A link between synaptic plasticity and reorganization of brain activity in Parkinson's disease
Source: Proc Natl Acad Sci U S A. 2021 Jan 11;118(3):e2013962118. doi: 10.1073/pnas.2013962118 (PMC7826364; doi:10.1073/pnas.2013962118)
Supplement: Supplementary File [file pnas.2013962118.sapp.pdf]

Supplementary Tables and Figures

Supplementary Table 1: Clinical and demographic characteristics of Parkinson 's disease (all, on- and off-medication) patients and healthy controls.

Characteristics	PD (n=14)	On-PD (n=7)	Off-PD (n=7)	HC (n=9)
Age	63.79 (5.57)	65.86 (5.36)	63.71 (5.09)	62.22 (4.49)
Gender (% female)	42.86	42.86	42.86	44.44
Education (years)	7.36 (4.75)	7.43 (4.83)	6.86 (4.38)	11.44 (4.80)
MMSE	28.86 (1.51)	28.57 (1.81)	29.14 (1.22)	29.56 (1.01)
GDS	7.00 (4.77)	8.57 (5.56)	5.43 (3.55)	4.89 (3.30)
UPDRS	19.00 (10.81)	15.71 (13.05)	22.29 (7.61)	
Hoehn-Yahr	1.50 (0.76)	1.43 (0.79)	1.57 (0.79)	
LEDD	760.43 (450.65)	982.86 (520.09)	538.00 (232.58)	

MMSE, Mini Mental State Examination; GDS, Geriatric Depression Scale; UPDRS, Unified Parkinson's Disease Rating Scale (section II-IV); LEDD, levodopa equivalent daily dose; On-PD: On medication PD; Off-PD: Off medication PD; HC: healthy controls. Patients were separated into off- and on-PD group, based on their medication status on the day of the functional assessment. Specifically, off-PD patients were deprived of their medication since the night before the experiment. In contrast, on-PD patients were allowed to take their medication on the night before and in the day of the experiment. Thus, UPDRS score reflects the median score of each of the above-mentioned groups and not the best on or worse off for all patients.

Supplementary Table 2: Between-group Slope and R Comparisons

Molecular-Molecular

		df	T-Statistics	p-value (slope)
**FEF vs Caudate**	**Left**	19	3.123	0.006
**FEF vs Putamen**	**Left**	19	2.223	0.039
**PEF vs Caudate**	**Right**	19	-0.225	0.824
**PEF vs Putamen**	**Right**	19	-1.702	0.105
			**Z score**	**p-value (R)**
**FEF vs Caudate**	**Left**		2.665	0.004
**FEF vs Putamen**	**Left**		2.038	0.021
**PEF vs Caudate**	**Right**		-1.023	0.153
**PEF vs Putamen**	**Right**		-1.998	0.023

Functional - Molecular

**Slope (Beta)**		**df**	**T-Statistics**	**p-value**
**FEF vs Putamen**	**Left**	19	4.369	0.0003
**FEF vs Putamen**	**Right**	19	2.643	0.016
**R**			**Z score**	**p-value (R)**
**FEF vs Putamen**	**Left**		3.518	0.0001
**FEF vs Putamen**	**Right**		2.515	0.006

Legend: Between group comparisons for regression slopes and R values (left and right hemispheres)

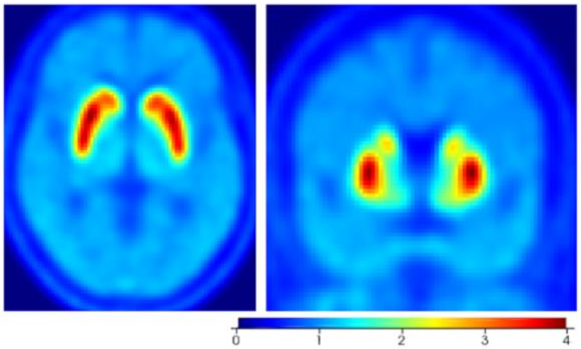

Supplementary Figure 1: D_2 receptor DVR map in healthy participants in transversal (left) and coronal (right) planes. This figure is a mean image of our healthy participants. Color bar: DVR ($mL.mL^{-1}$).

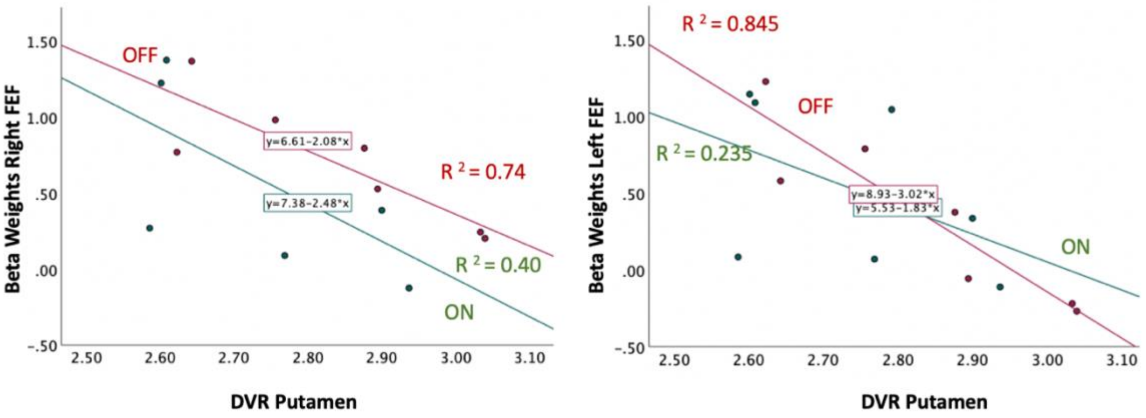

Supplementary Figure 2: Multimodal Molecular-functional correlations in PD patients on and off-medication. Relationship between DVR in the putamen and the beta weights in the left FEF and DVR in the putamen and beta weights in the right FEF during vertical PS, for PD participants on and off medication. Trendline, correlation coefficient and p-value are presented. The dashed lines represent the 95% confidence band.
